# Short and Long Term Variation in Ultraviolet Radiation and Multiple Sclerosis

**DOI:** 10.3390/ijerph9030685

**Published:** 2012-02-24

**Authors:** Cristina Menni, Walter E. Lowell, Joan Bentzen, Roberto Bergamaschi, Filippo Martinelli Boneschi, Vittorio Martinelli, Luisa Bernardinelli, Egon Stenager, George E. Davis, Luisa Foco

**Affiliations:** 1 Department of Applied Health Sciences, University of Pavia, Via Bassi 21, 27100 Pavia, Italy; Email: L.Bernardinelli@statslab.cam.ac.uk (L.B.); luisa.foco@unipv.it (L.F.); 2 Psybernetics Research Group, 04330 Augusta, Maine, USA; Email: welowell@gmail.com (W.E.L.); georgedavi@gmail.com (G.E.D.); 3 The Danish Multiple Sclerosis Registry, National Institute of Public Health, 1353 Copenhagen, Denmark; Email: joab@si-folkesundhed.dk (J.B.); Egon.Stenager@shs.regionsyddanmark.dk (E.S.); 4 Neurological Institute Casimiro Mondino, Interdepartment Research Center for Multiple Sclerosis, 27100 Pavia, Italy; Email: roberto.bergamaschi@mondino.it; 5 Department of Neuro-Rehabilitation, Scientific Institute San Raffaele, 20100 Milan, Italy; Email: martinelli.filippo@hsr.it (F.M.B.); martinelli.vittorio@hsr.it (V.M.); 6 Department of Pure Mathematics and Mathematical Statistics, University of Cambridge, CB3 0WA Cambridge, UK; 7 Institute of Regional Health Services, University of Southern Denmark, 5000 Odense, Denmark; 8 MS-Clinic of Southern Jutland (Sonderborg, Vejle, Esbjerg), Department of Neurology, 6400 Sonderborg, Denmark

**Keywords:** multiple sclerosis, seasonality, solar cycles, variation in ultraviolet radiation

## Abstract

We examined the role of ultraviolet radiation (UVR) in persons diagnosed with multiple sclerosis (MS) in four different populations, Italians, Danish, White and African Americans. We tested whether variation in UVR as determined by seasons (short term variation) and solar cycles (long term variation) is related to MS birth month and to survival as measured by lifespan. Cases were selected from three Italian MS Case Registries (2,737); from the United States National Center for Health Statistics (56,020); and from the Danish Multiple Sclerosis registry (15,900). Chi-square tests were used to study the pattern of month of birth distribution in patients with MS comparing with general population data. T-tests were employed to study solar cycles association with lifespan. A surplus of births was observed in June for White Americans. A decrease of births in October and November, though not significant after multiple testing correction, was observed in the three populations. In White American with MS overall, males and females, we found that solar cycle is associated with lifespan. We found that season and solar cycles have some role in MS susceptibility and life duration. However, this is an exploratory analysis and further work is needed to discern the association.

## 1. Introduction

Solar radiation not only supplies the biosphere with energy and synchronizes circadian rhythms, but may also actively modify genomes, both through mutation and epigenetic mechanisms [1]. Many epidemiological studies report that some diseases occur with higher incidence in persons born in particular months, referred to as seasonality [2]. This suggests that an environmental factor may operate during gestation. Seasonal pattern of birth have been observed for schizophrenia and bipolar disorders [3], and for some autoimmune diseases, as type 1 diabetes [4], Crohn’s disease [5], myositis [6] and celiac disease [7] among others. 

Seasonality effects have also been found for multiple sclerosis (MS) [8], a chronic inflammatory autoimmune disease of the central nervous system, which causes myelin destruction and neuronal cell degeneration leading the patient to a progressive disability. The disease has a higher incidence in females than in males and most commonly presents in the second to fourth decade of life. The cause of MS is unknown, but it most likely results from a complex interplay of both genetics and environmental factors. While genetic factors shape the overall population susceptibility, epidemiological studies suggest an important role of the environment in disease initiation and modulation [9]. Findings on seasonality effects, however, are not consistent. A Canadian study using data from Canada and UK and combined with previously reported data from Denmark and Sweden [8] found a statistically significant difference in seasonal pattern of births among MS patients, but to estimate the numbers of MS cases born in a particular month, the authors summed number of births over the years 1926–1970 without taking into account year of birth. It has been argued [10] that biases could have arisen as MS cases born in one year were weighted relative to persons with completely different follow-up time. Another Canadian study [11] was unable to detect any significant seasonal difference. In Sicily, an excess in birth of MS patients occurred in the summer, with a peak in August [12]. A trend was also seen in a recent study on 1524 Australian patients with excess risk for those born in November-December, which is equivalent to April–May in the Northern hemisphere [13].

A relationship between the geographical distribution of MS, exposure to sunlight, and hypovitaminosis D has long been advocated [14]. Scientists have hypothesized that MS is rare in the tropics because people synthesize great quantities of vitamin D from exposure to ultraviolet radiation (UVR) in equatorial sunlight. On the contrary, MS is more prevalent in the high latitudes of Northern Europe and America where there is less exposure to sunlight [15]. Sardinia is an exception to this rule possibly due to inbreeding and genetic drift. Indeed, it has a high UVR exposure and a high MS prevalence comparable to that of Northern Europe [16]. These arguments support the so called “latitude hypothesis”, *i.e.*, vitamin D may protect against the development of MS [17]. Prevalence of MS is lower than expected at high latitude where vitamin D intake is high because of fatty fish consumption [18]. Also, MS seems to decrease with migration from an higher to a lower latitude [19]. However, as emphasized by two recent reviews, a direct cause-and-effect relationship between vitamin D deficiency and MS has not yet been established thus suggesting that UVR may have effects beyond making vitamin D [18,20,21].

Davis and Lowell reported the association between intensity of solar radiation, referred as solar cycles, and various diseases [22]. The term “solar cycle” refers to the periodic rise and fall of the intensity of solar radiation where the Sun increases its irradiance on average every 11 years (range 9–14). Sunspots, the manifestation of magnetic storms on the Sun’s surface, are proxies for increase radiation intensity and have been recorded for centuries. During cycle peaks, sunspots increase in number and size with a concomitant increase in radiation [23]. In particular, the solar cycle variation causes as much as a 400% variation in UVB at 300 nm reaching the earth [24]. Relatively small changes in solar radiation (0.1%), due to solar cycle variability, may also significantly modify regional surface temperatures [25].

These results and those regarding seasonality, support the notion that UVR is instrumental in modifying the human genome not only by overt mutation DNA, but also by an epigenetic mechanism [1]. This hypothesis is supported by the recent work of Feng *et al.* showing that binding of histone deacetylase 3 (HDAC3) to the genome depends on light circadian rhythm: thus light can actually contribute to chromatin remodeling, modifying gene expression [26]. UVR significantly affects DNA and the effects are cumulative throughout life [27]. Epigenetic mechanisms are important as they enable the embryo to quickly modify its genetic library to match current environmental conditions and hence to maximize survival. Human lifespan may be modulated by sunlight as there is a statistically significant variation in lifespan by month of birth between peaks and non-peaks of solar cycles [23].

The aim of this study is to examine the role of UVR in persons diagnosed with MS in four different populations, Italians, Danish, and White and African Americans. In particular we test whether variation in UVR as determined by seasons (short term variation) and solar cycles (long term variation) is related to MS birth month and to survival as measured by lifespan.

## 2. Experimental Section

### 2.1. Study Populations

*Italy*: Demographical data of MS patients were collected from the Case Registry of the Division of Neurology of the Nuoro Hospital, Sardinia (n = 890); from the Case Registry of the Interdepartment Research Center for Multiple Sclerosis, Neurological Institute Casimiro Mondino, Pavia (n = 683); and from the Case series from San Raffaele Scientific Institute (ISR), Milan (n = 1164). The three registries are ongoing. They were started respectively in 1995, 1990 and 1996.

For this study, all records include birth year (YOB), birth month (MOB) and gender. National and regional data of month/year specific birth were retrieved from the Italian Statistical Institute (ISTAT) database. We normalized for the Italian general population data as the MOB distribution in the three studied Italian areas did not differ significantly. All Italian patients were of Caucasian origins.

*USA*: Death records were collected from the United States National Center for Health Statistics (NCHS) from 1979 to 2005 which contains 59 million death records. ICD9 code 340 and ICD10 code G35 for cause of death were used to extract records for MS (N = 56,020) from the NCHS data set. Data used in this sample included sex, ICD9 & ICD10 cause of death, state of birth, month and year of birth, month and year of death and ethnicity. National month/year specific birth was calculated using the NCHS dataset and pulling out the MS cases. As U.S. cases presented a great ethnic diversity (White, African American, American Indian, Asian and others), the analysis was restricted to the two largest ethnic groups: White and African Americans. White and African Americans were analyzed separately throughout because they respond differently to solar UVR. African American people have a different skin pigmentation and they more efficiently block UVB. As a consequence, they tend to be more vitamin D deficient. This unexpectedly inversely correlates with MS risk [18]. 

*Denmark*: Cases were selected out of 15,900 cases from the Danish Multiple Sclerosis registry [28]. The registry was established in 1948 with a nationwide prevalence survey and has since then collected information on MS patients from multiple sources. Virtually all Danish residents in whom multiple sclerosis was diagnosed by a neurologist or in a department of neurology are registered [29]. The registry is continuously updated with new information on registered cases and new cases. For the seasonality analysis, cases were selected to have a definite diagnosis of MS before 2005; while for the solar cycle analysis, deceased MS patients were chosen. National month/year specific birth were extracted from Statistics Denmark [30]. Almost all Danish cases were of Caucasian origin [31].

### 2.2. Solar Data

The average number of annual sunspots per year was collected from the U.S. Department of Commerce National Oceanic and Atmospheric Administration (NOAA) web site [32] and the three peak years of sunspots of each of the past twelve cycles was obtained. Solar cycles MAX years are 1905, 1906, 1907, 1917, 1918, 1919, 1927, 1928, 1929, 1937, 1938, 1939, 1947, 1948, 1949, 1957, 1958, 1959, 1968, 1969, 1970, 1979, 1980, 1981, 1989, 1990, 1991, 2000, 2001, 2002. Solar cycles MIN years are 1895–1904, 1908–1916, 1920–1926, 1930–1936, 1940–1946, 1950–1956, 1960–1967, 1971–1978, 1982–1988, 1992–1999, 2003–2005.

### 2.3. Seasonality Analysis

For the seasonality analysis, our hypothesis was that people with MS might show a different pattern of month of birth. For Italy and Denmark, expected cases were calculated based on the MOB of each general population born in the same years as the cases. The U.S. population based controls came from the 59 million death records and include all non-MS cases. Expected values for White and African Americans were calculated differently based on the birth distribution of White and African Americans in the general population. Chi-square statistics were calculated in 2 × 2 tables with the MOB in question against the remaining 11 months. We accounted for multiple testing using Bonferroni correction.

### 2.4. Solar Cycles Analysis

Age at death was calculated from month and year of birth to month and year of death. To examine the influence of solar cycles on survival, birth year data were grouped by solar maximum or solar minimum defined as follows: the year before and the year after the peak were defined as the Maximum Solar Period (MAX); the years before and after each three year MAX cycle were grouped as Minimum Solar Period (MIN). T-tests were use to determine whether the difference between MAX and MIN groups was due to chance variation.

## 3. Results and Discussion

The gender distribution of the MS study populations is reported in Table 1. The female:male ratio varies in the different populations with the Italians having the highest (the number of females with MS is more than twice the number of males) and the Danish the lowest (the number of females with MS is a bit more than one and a half the number of males). However, as expected, the number of affected females always exceeds the number of affected males.

**Table 1:** Gender distribution of the MS study populations, n (%).

Sex	Italians	Danish	White Americans	African Americans
**F**	1,897 (69)	9,856 (62)	32,456 (64)	3,630 (67)
**M**	840 (31)	6,044 (38)	18,194 (36)	1,740 (32)
**F:M**	2.26	1.63	1.78	2.09
**TOTAL**	2,737	15,900	50,650	5,370

### 3.1. Seasonality Analysis

We compared the number of individuals with MS born in each month *versus* the other 11 months. The expected number of births was calculated using the distribution of birth in the general population for Italy and Denmark (Table 2), and of population controls for the U.S. data (Table 3).

**Table 2:** Seasonality overall results for Italy and Denmark normalized for the specific general population data.

	**Italy**					**Denmark**				
**MOB**	**Obs**	**Exp**	**Obs/Exp**	**Month specific χ^2^**	***p* value**	**Obs**	**Exp**	**Obs/Exp**	**Month specific χ^2^**	***p* value**
			**(95% CI)**					**(95% CI)**		
January	248	231	1.07[0.94; 1.21]	1.31	0.2531	1274	1304	0.98 [0.92;1.03]	0.75	0.3864
February	204	204	1.00[0.86; 1.14]	0	0.9907	1240	1274	0.97[0.92;1.03]	0.99	0.3197
March	241	219	1.10[0.96; 1.24]	2.49	0.1147	1523	1462	1.04[0.99;1.09]	2.8	0.0942
**April**	238	203	1.17 [1.02; 1.32]	6.36	**0.0117**	1478	1420	1.04[0.99;1.09]	2.6	0.1068
May	228	234	0.97[0.85; 1.1]	0.17	0.684	1451	1417	1.02[0.97;1.08]	0.9	0.3427
June	242	221	1.10[0.96; 1.23]	2.2	0.1377	1384	1326	1.04[0.99;1.1]	2.77	0.0960
July	220	240	0.92[0.8; 1.04]	1.75	0.1863	1322	1339	0.99[0.93;1.04]	0.24	0.6242
August	216	235	0.92[0.79; 1.04]	1.76	0.1845	1358	1328	1.02[0.97;1.08]	0.74	0.3896
September	243	251	0.97[0.85; 1.09]	0.26	0.6135	1263	1310	0.96[0.91;1.02]	1.84	0.1749
**October**	219	250	0.88[0.76; 0.99]	4.15	**0.0416**	1243	1267	0.98[0.93;1.04]	0.49	0.4839
**November**	208	224	0.93[0.80; 1.06]	1.22	0.27	1139	1208	0.94[0.89;1]	4.27	**0.0389**
December	230	226	1.02[0.89; 1.15]	0.08	0.77251	1225	1246	0.98[0.93;1.04]	0.38	0.5376
	*chisq(11) = 19.95, p =0 .0461*					*chisq(11) = 17.19, p =0.1023*				

**Table 3:** Seasonality overall results for U.S. data normalized for the population based controls.

	White Americans					African Americans				
MOB	Obs	Exp	Obs/Exp (95% CI)	Month specific χ^2^	*p* value	Obs	Exp	Obs/Exp (95% CI)	Month specific χ^2^	*p* value
January	4178	4291	0.97[0.94;1]	3.251	0.07137	458	452	1.01[0.92;1.1]	0.087	0.7681
February	3894	4034	0.97[0.94;1]	5.279	**0.021582**	403	431	0.94[0.85;1.03]	1.978	0.1596
March	4405	4402	1[0.97;1.03]	0.002	0.962259	460	480	0.96[0.87;1.05]	0.915	0.33876
April	4033	4069	0.99[0.96;1.02]	0.346	0.556199	423	441	0.96[0.87;1.05]	0.8	0.37097
May	4152	4140	1[0.97;1.03]	0.038	0.845688	411	443	0.93[0.84;1.02]	2.519	0.1125
June	4255	4027	1.06[1.03;1.09]	14.024	**0.000181**	425	424	1[0.9;1.1]	0.003	0.9596
July	4480	4318	1.04[1.01;1.07]	6.644	**0.009948**	504	453	1.11[1.01;1.21]	6.271	**0.0123**
August	4537	4480	1.01[0.98;1.04]	0.796	0.372414	494	481	1.03[0.94;1.12]	0.386	0.5345
September	4425	4416	1[0.97;1.03]	0.016	0.8997	463	464	1[0.91;1.09]	0.002	0.96
October	4175	4312	0.97[0.94;1]	4.758	**0.0291**	483	441	1.1[1;1.2]	4.358	**0.037**
November	4030	4016	1[0.97;1.03]	0.053	0.8179	394	407	0.97[0.87;1.07]	0.449	0.5027
December	4086	4145	0.99[0.96;1.02]	0.915	0.33888	452	454	1[0.91;1.09]	0.01	0.92
	*chisq(11) = 33.16, p < 0.0001*					*chisq(11) = 16.30, p = 0.1304*				

In the Italian population (Table 2) we found a significant surplus of births in April (χ^2^ = 6.36, uncorrected *p* = 0.0117) and a lower incidence in October (χ^2^ = 4.15, uncorrected *p* = 0.0416) (Table 2), but these were not significant after multiple testing correction. When we analyzed males and females separately normalizing for males and females general population data, we found an excess of birth in April for Italian men (χ^2^ = 5.64, uncorrected *p* = 0.0175) and a decrease of birth in October for Italian women (χ^2^ = 5.02, uncorrected *p* = 0.025). However, these were not significant after multiple testing correction.

In the Danish sample, a lower incidence was observed among people born in November (χ^2^ = 4.27, uncorrected *p* = 0.0389), but again this did not pass multiple testing correction (Table 2). We could not perform the analysis by sex as gender of the general population, according to MOB, was not available on the Statistic Denmark database.

In the U.S. population, June and July had significantly more cases than expected for White Americans (June: 5.7% more, χ^2^ = 14.024, uncorrected *p* = 0.000181 or *p* = 0.002172 with Bonferroni correction; July: χ^2^ = 6.644, uncorrected *p* = 0.0099), while October had significantly less (χ^2^ = 4.758, uncorrected *p* = 0.0291). Only June was significant also after accounting for multiple testing using Bonferroni correction (Table 3). A surplus of birth in July was also seen in African Americans (χ^2^ = 6.271, uncorrected *p* = 0.0123), though not significant after multiple testing correction and an excess of birth was observed in October (χ^2^ = 4.358, uncorrected *p* = 0.037). 

When stratifying by sex, we confirmed a statistical significant increase in births in June and July for White American females (June: 6.2%, χ^2^ = 10.58, uncorrected *p* = 0.001146 or *p* = 0.013752 with Bonferroni correction; July: χ^2^ = 4.89, uncorrected *p* = 0.027059), and a significant increase in births in May for white American males (χ^2^ = 4.39, uncorrected *p* = 0.0361) and a decrease of birth in October for white American males (χ^2^ = 4.3, uncorrected *p* = 0.0382). Again, June only was significant after accounting for multiple testing correction. A surplus of birth was also observed in African American females (χ^2^ = 6.02, uncorrected *p* = 0.014168), while no difference was found in African American men.

### 3.2. Solar Cycles Analysis

From the NOOA database, the average annual sunspot number for the past 250 years is 49; for the past 60 years the average is 107.5; for the most powerful cycles (sunspots >135), the average is 154, about three times the 250-year average. Also, the average annual sunspot number in a MAX year is 107.71 (SD = 43.33), while the average annual sunspot number in a MIN year is 38.73 (SD = 33.60). It was not possible to perform a solar cycle analysis on the Italian cohorts as the majority of the patients (>99%) were still alive.

We found a statistically significant association in White Americans (Table 4): those born in solar cycles MAX years have a significantly shorter lifespan (−0.64 year, *p* ≤ 0.0001) than those born in solar cycles MIN years. When we stratified by sex (Table 5), we saw similar results for both White American males (−0.5 year, *p* = 0.0184) and females (−0.71 year, *p* = 0.0006). No significant difference emerged neither in the African American sample nor in the Danish population.

**Table 4:** Counts and Mean age at death by solar Max and solar MIN for the MS cohort data.

	White Americans		African Americans		Danish	
**RGRP**	*N*	*Mean (SD)*	*N*	*Mean (SD)*	*N*	*Mean (SD)*
**MAX**	15,054	59.43 (13.26)	1,591	51.68 (13.26)	1,974	60.09 (13.60)
**MIN**	35,596	60.07 (13.67)	3,779	51.57 (13.75)	5,195	60.26 (13.76)
		*p* ≤ *0.0001*		*p = 0.7849*		*p = 0.6517*

**Table 5:** Mean age at death for the MS cohort by sex.

	Males						Females					
	White Americans		Black Americans		Danish		White Americans		Black Americans		Danish	
**RGRP**	*N*	*Mean (SD)*	*N*	*Mean (SD)*	*N*	*Mean (SD)*	*N*	*Mean (SD)*	*N*	*Mean (SD)*	*N*	*Mean (SD)*
**MAX**	5,440	58.82 (12.74)	521	51.45 (13.22)	889	59.25 (13.09)	9,614	59.77 (13.53)	1,070	51.79 (13.28)	1,085	60.78 (13.41)
**MIN**	12,754	59.32 (13.35)	1,219	51.36 (13.86)	2,337	59.30 (12.94)	22,842	60.48 (13.83)	2,560	51.67 (13.70)	2,858	61.04 (13.72)
	***p = 0.0184***		*p = 0.9009*		*p = 0.9340*		***p < 0.0001***		*p = 0.7991*		*p = 0.6025*	

### 3.3. Discussion

In this paper, we first show that there is a significant pattern of risk of MS with MOB. Among White Americans with MS, 5.7% more than expected were born in June. This finding is confirmed in White American females where the percentage increases to 6.2%. A similar trend, though not significant after accounting for multiple testing, was found in White American men with a peak in May, for Italians with a peak in April and for Italian men with a peak again in April. A trend of lower risk of developing MS was observed in those born in October (White Americans: overall and males; Italians: overall and females) and in November (Danish). MOB October through November *versus* May through July mirrors months of conception January through February and August through October suggesting that increasing and decreasing UVR at conception has an effect on MS. This was also reported in a recent Australian study [13] where low maternal exposure to UVR at conception was associated with a higher risk of MS for the offspring. Moreover, an increase of births in April was seen in a French study [33], even though it did not reach statistical significance and in a pooled analysis of data from Canada, UK, Denmark and Sweden an excess of birth was reported in May and a decrease in November [8]. 

In the U.S. sample, we found that there is a statistically significant difference between MAX and MIN solar cycle years for White Americans (overall, males and females). We were unable, however, to replicate this finding both on African Americans and in the Danish sample (Table 4). The reason for this could be that the difference in lifespan between those born in solar cycles MAX and MIN year is relatively small and hence it only reached the statistical significance in the U.S. sample, as it is a large cohort. 

A recent paper [34] about the MS genome in identical twins underscores that genetics alone do not explain the phenotype of MS; rather, an important environmental factor must be in play. We think that it may be UVR itself, possibly the ultraviolet range, in those that inherit a particularly sensitive sex-linked genome where females are twice as likely as males to develop MS (as confirmed in Table 1) because of their increased sensitivity to long-term changes in sunlight (Table 5). Increased sensitiveness of females to solar cycles could be explained considering the role of sunlight in vitamin D metabolism and the link between vitamin D and estrogen metabolism. Indeed, calcitriol, a biologically active compound derived from vitamin D, regulates the expression of the Cyp19, an aromatase necessary to synthesize estrogens [35,36,37]. Thus sunlight-driven variations in vitamin D level could also influence estrogen levels.

Our study has some strengths. First, the Danish sample originates from a close to complete registry of a whole country with registration of cases in the last 60 years. Second, the U.S. sample is drawn from the NCHS, which contains more than 99% of all births and deaths registered in the U.S. Third, we had data on four different populations and hence the chance of comparing the effect of sunlight on people with different geographical and ethnic background. Moreover, previous studies of autoimmune diseases including celiac disease and myositis, show that birth season differs based on race or ethnicity [6,7]. We had information of ethnic background and were therefore able to run a stratified analysis. 

Our study also has some limitations. First, as the U.S. data is drawn from the NCHS and as there are more people dying with MS rather than of MS, we may have missed some cases. Indeed, a Danish study found that more than half (56.5%) MS patients have MS as cause of death on the death certificate [38]. It is likely that those excluded were patients suffering from a less severe MS (*i.e.*, not severe enough to die from it). Second, we could not run the solar cycle analysis on the Italian data as the majority of the Italian patients were still alive. Third, the statistical analysis employed in the paper does not adjust for factors such as variation in viral outbreaks, epidemics, other seasonally varying factors such as ambient temperature, changes in diet, temporal trends, *etc*. and thus does not rule out the possibility that such factors might explain variation in season of birth or in lifespan based on year of birth. Also, it is impossible to untangle whether the observed surplus of births in June and July in the U.S. data, is due to the fact that MS is more likely to occur in those born in those months or if there is something about being born in those months that increases the risk of death. 

Finally, we recognize that we compared three different datasets with different methods of collecting data and that there are large methodological problems in doing so. However, this is an exploratory analysis. 

## 4. Conclusions

We think that our results are novel and interesting. To our knowledge, we are the first to investigate long term UVR variation, measured as the influence of solar cycles, on MS. Even if the results are not replicated in all the population studied, we did find a significant signal in White American males and females and we believe this is worth reporting. Moreover, we confirm that there is a seasonality effect on MS suggesting that there is some environmental factor operating during gestation.

The next steps would be to investigate (i) whether the difference in lifespan we observe for MS patients differs from other causes of death in magnitude; and (ii) whether people born in solar cycles MAX years differ for some genomic features from those born in solar MIN year. Assuming that solar radiation affects the human genome through mutation and epigenetic mechanisms, a case-only genome wide study of genetic and methylation profiles, comparing groups of MAX and MIN MS patients, could identify MS epigenetic susceptibility factors induced by exposure to solar radiation and genetic profiles ideally referable to vitamin D metabolism pathways.
